# Association of Circulating Heme Oxygenase-1, Lipid Profile and Coronary Disease Phenotype in Patients with Chronic Coronary Syndrome

**DOI:** 10.3390/antiox10122002

**Published:** 2021-12-15

**Authors:** Chiara Caselli, Raffaele De Caterina, Rosetta Ragusa, Riccardo Liga, Alessia Gimelli, Arthur J. H. A. Scholte, Aldo Clerico, Juhani Knuuti, Danilo Neglia

**Affiliations:** 1 Institute of Clinical Physiology, National Research Council (IFC-CNR), Via Moruzzi, 1, 56124 Pisa, Italy; rragusa@ifc.cnr.it; 2 Fondazione Toscana G. Monasterio, 56124 Pisa, Italy; gimelli@ftgm.it (A.G.); aldoclerico1948@gmail.com (A.C.); dneglia@ftgm.it (D.N.); 3 Institute of Cardiology, University of Pisa, 56124 Pisa, Italy; raffaele.decaterina@unipi.it (R.D.C.); riccardo.liga@gmail.com (R.L.); 4 Scuola Superiore Sant’Anna, 56124 Pisa, Italy; 5 Department of Cardiology, Leiden University Medical Center, 2333 Leiden, The Netherlands; a.j.h.a.scholte@lumc.nl; 6 Turku University Hospital, University of Turku, 20520 Turku, Finland; juhani.knuuti@tyks.fi

**Keywords:** Heme Oxygenase-1 (HO-1), oxidative stress, coronary artery disease

## Abstract

Background. The NF-E2–related factor 2 (Nrf2)/Heme Oxygenase-1 (HO-1) pathway has an emerging role in atherosclerosis. Activated by oxidative stress, it is deemed to exert athero-protective effects. We aimed at evaluating the relationships between plasma HO-1, clinical/molecular profiles and coronary disease patterns in patients with chronic coronary syndromes (CCS). Methods. HO-1 was measured in 526 patients (60 ± 9 years, 318 males) with CCS. Coronary computed tomography angiography (CTA) and stress imaging were used to assess the disease phenotype (coronary atherosclerosis and myocardial ischemia) in a subgroup of 347 patients. Results. In the overall population, HO-1 median value (25–75 percentile) was 5.195 (1.75–8.25) ng/mL. Patients with higher HO-1 were more frequently male, had a higher BMI and lower LVEF%, but otherwise similar risk factors than the other patients. Their bio-humoral profile was characterized by higher markers of endothelial/myocardial dysfunction, but lower levels of cholesterol lipoproteins. Coronary artery disease was characterized by more diffuse atherosclerosis, with mainly non-obstructive and calcified plaques, and a higher prevalence of functional ischemia. Conclusion: In patients with CCS, higher plasma HO-1 levels are associated with lower cholesterol and a more diffuse but mainly non-obstructive coronary atherosclerosis, confirming a potential role for the Nrf2/HO-1 pathway as a protective feedback.

## 1. Introduction

Cardiovascular disease (CVD) accounts for the largest proportion of deaths in Western Countries [1]. Atherosclerosis, the main underlying pathological basis for CVD, is a chronic disease with complex pathogenesis [2], with endothelial dysfunction, inflammation, lipid deposition, and oxidative stress involved in the initiation and progression of atherosclerosis [3,4,5]. Antioxidant defenses reported to be associated with atherosclerosis include the activation of nuclear erythroid factor 2–related factor 2 (Nrf2)/Heme Oxygenase-1 (HO-1) pathway [6,7,8]. In unstressed state, Nrf2 is constitutively expressed and blocked by Kelch-like erythroid cell-derived protein with cap ‘n’ collar homology-associated protein 1 (Keap1) in the cytoplasm, causing its degradation by proteasomes [7,8]. In condition of oxidative stress, the complex Keap1/Nrf2 separates and Nrf2 transfers into the nucleus. Thus, Nrf2 binds the antioxidant responsive element (ARE) and induces the transcription of proteins with antioxidant actions [7,8]. Among them, HO-1 has a pivotal role in the antioxidant mechanism within the cell, promoting the degradation of the prooxidant heme to carbon monoxide, biliverdin, and ferrous ion [7,8]. HO-1 is involved in protective mechanisms in several pathological conditions, including endothelial dysfunction, inflammation, atherosclerosis, and myocardial ischemia/reperfusion injury [9,10,11,12,13]. In animal models, a lack of HO-1 was shown to accelerate atherosclerosis, whilst HO-1 stimulation promoted atherosclerosis reduction [14]. In human studies, alterations of the HO-1 gene are variably associated with coronary artery disease [15,16]. In clinical populations, circulating HO-1 levels have been associated with various atherosclerotic diseases [9]. All of these data support the notion that HO-1 may play a protective role against progression of atherosclerotic disease. Nevertheless, the relationship between circulating HO-1 levels, other atherosclerotic risk profiles and vascular disease phenotype has not been fully explored. 

The present study aimed at evaluating the possible associations of HO-1 circulating plasma levels with clinical and bio-humoral atherosclerotic risk profiles, including metabolic, inflammatory and organ function biomarkers, in a prospectively enrolled population of patients with chronic coronary syndrome (CCS) from the European Evaluation of Integrated Cardiac Imaging (EVINCI) study [17]. In particular, the relationship of HO-1 levels with the coronary disease phenotype was assessed in patients in whom the presence, extent and severity of coronary atherosclerosis and ischemia were evaluated by coronary computed tomography angiography (CTA) and stress cardiac imaging. 

## 2. Material and Methods

### 2.1. Study Design and Population

Design and primary results of the EVINCI study have been previously reported (http://www.clinicaltrials.gov, NCT00979199, 21 July 2014) [17]. According to the protocol, each patient with CCS had to perform a non-invasive imaging diagnostic work-up, including coronary CTA and/or stress imaging (perfusion or function evaluation). Blood samples were collected before non-invasive imaging, and plasma aliquots were stored in the EVINCI Bio-Bank. Ethics Committee approval was provided by each participating center (Ethics Committee approval number of the EVINCI Coordinating Center, IFC-CNR: 2719/2009, 12 February 2009), and all subjects provided written informed consent. 

Over the 697 patients initially enrolled in EVINCI, the population of the present study includes 526 patients in whom plasma levels of HO-1 were evaluated (clinical population), of whom 347 underwent both CTA and stress imaging (imaging population). 

### 2.2. Clinical Definitions

Diabetes was defined as fasting plasma glucose (FPG) >125 mg/dL or treatment. Homeostatic model assessment of insulin resistance index (HOMA-IR) was calculated as fasting glucose (mg/dL) × fasting insulin (pmol/L)/8.66. Body Mass Index (BMI) was calculated as body weight (in kg) divided by the square of the height (in m). The presence of metabolic syndrome was diagnosed as previously defined [18]. 

### 2.3. HO-1 Measurements and Bio-Humoral Profile

HO-1 plasma levels were measured in the available blood samples stored in the EVINCI biological bank (IFC-CNR, Pisa, Italy) by a dedicated ELISA (Enzo Life Science, Farmingdale, NY, USA). In order to complete the bio-humoral profile of the study patients, additional biomarkers, involving oxidative stress, glucose and lipid profiles, adipose tissue, hepatic, cardiac and renal function, and remodeling and inflammation, were measured using standard methods, as previously reported [19]. 

### 2.4. Non-Invasive Imaging 

Patient preparation, cardiovascular stress, administration of radiopharmaceutical or contrast medium, image acquisition and quality control for each non-invasive imaging technique followed standard protocols, based on best available clinical practice. Image analysis and interpretation were performed for each modality at specific core labs by dedicated observers blinded to the clinical data and to any other test results [17]. 

#### 2.4.1. Coronary CTA

CTA acquisition and analysis protocols have been previously reported in detail [17,20]. Interpretable coronary segments (AHA 17-coronary segment model) were classified according to the degree of stenosis in three different categories: normal, non-obstructive (in presence of <50% stenosis), and obstructive (for stenoses >50%). Plaque composition was visually classified as calcified, non-calcified, or mixed. 

A previously validated CTA score, used as an indicator of the global coronary atherosclerotic burden and risk, was derived in each patient by integration of all data on the location, severity and composition of plaques [20]. Agatston CAC score was computed according to standard methods.

#### 2.4.2. Non-Invasive Stress Imaging Analysis 

Stress myocardial perfusion imaging tests were defined as abnormal if there was either an inducible perfusion abnormality or myocardial scarring. Each of the 17 myocardial segments was classified according to the following perfusion scores: 0 = normal, 1 = mild reduction, 2 = moderate reduction, 3 = severe reduction or 4 = absent. The segmental scores were summed for the stress and rest images, and a summed difference score (SDS) was calculated as the difference between stress and rest summed scores. Inducible myocardial ischemia was defined as an inducible perfusion defect with a SDS ≥2. Scarring was defined similarly to the summed segmental rest score. For stress myocardial wall motion imaging tests, segmental wall motion was scored at rest and during stress as normal (0), hypokinetic (1), akinetic (2), or dyskinetic (3). Inducible myocardial ischemia was defined as an increase in segmental wall motion score ≥ 1 from rest to stress in at least two contiguous segments. Scarring was defined similarly from the resting wall motion score.

### 2.5. Statistical Analysis

Categorical variables are presented as numbers (percentage), continuous variables as mean ± SD. Not normally distributed variables underwent logarithmic transformation. Patients were subdivided into two groups according to HO-1 median value (5.20 ng/mL): “low HO-1” group, including patients with HO-1 values lower that the median, and “high HO-1” group, including those with HO-1 value higher than the median value.

Clinical and bio-humoral variables were compared among patients with lower and higher HO-1 plasma levels using Student’s *t*-test or the Chi-square test, as appropriate. Linear regression was used to estimate the effect of clinical and bio-humoral variables on HO-1 levels. The multivariate model was developed considering variables with a *p* value < 0.1 at univariate analysis, and then using backward and forward stepwise selections to build up the final model. 

Coronary CTA and stress imaging features were also compared among patients with lower and higher HO-1 plasma levels using Student’s *t*-test or the Chi-square test, as appropriate. Coronary plaque features (non-obstructive vs. obstructive; calcified vs. mixed/non-calcified) were compared among patients subdivided according to HO-1 quartiles. Multiple comparison was performed by ANOVA test using post-hoc analysis.

Clinical and bio-humoral variables were compared according to the presence of coronary atherosclerosis with or without inducible ischemia. Again, multiple comparison was performed by ANOVA test using post-hoc analysis.

All analyses were performed using the SPSS 23 software. A two-sided value of *p* < 0.05 was considered statistically significant. There is no multiplicity adjustment implemented in statistical testing.

## 3. Results

### 3.1. HO-1 Plasma Levels, Clinical and Bio-Humoral Profiles

Demographic and clinical characteristics, cardiovascular risk factors, and medication use of the clinical population are detailed in Table 1. Mean age was 60 ± 9 years and 60% of patients were male. The frequency of typical chest pain was 26%, and the mean value of LVEF% was 60 ± 8. Family history of CAD was present in 35% of patients and the majority of the population had hypercholesterolemia (60%) and hypertension (66%). Diabetes was diagnosed in 177 (34%), and metabolic syndrome in 181 (34%) patients. 

When patients were divided in two groups according to the HO-1 median value, the frequency of male gender and of the metabolic syndrome, as well as BMI values, were significantly higher in the “high HO1” group, while the mean value of LVEF% was significantly lower. No difference in medications use was observed among HO-1 groups.

The comparison of bio-humoral measurements between the two groups is reported in Table 2. Patients with “high HO-1” showed higher levels of GGT, a marker of oxidative stress. No differences were observed for the glucose profile, while the lipid profile was characterized by significant lower levels of Total-C, LDL-C, HDL-C, ApoB, Lp (a) and PCSK9 in the “high HO-1” group compared with the “low HO-1” group. Plasma levels of ApoA1 were not different between HO-1 groups, while significantly higher values of ApoA1/HDL-C and lower of ApoA1/ApoB were observed in patients with “high HO-1” than in patients with “low HO-1”. Adiponectin, a marker of adipose tissue function, as well as MMP-9 and ALP, markers of remodeling, were lower, but the inflammatory cytokine IL-6 was higher in patients with “high HO-1”. Among cardiac biomarkers, only hs-cTnI was higher in patients with “high HO-1”, together with creatinine, a marker of renal function. 

Among clinical and bio-humoral variables significantly associated with HO-1 plasma levels at univariate analysis (Tables S1 and S2 of Supplementary Materials), an independent and negative association of age, HDL-C/Apo A1, Apo B and Lp (a) levels as well as an independent and positive association of nitrate use, IL-6, and hs-cTnI levels with HO-1 values was observed at multivariate analysis (Table 3). These results were confirmed when patients were divided in groups according to HO-1 quartiles: age, HDL-C/Apo A1, Apo B, and Lp (a) showed a trend to decrease across HO-1 quartiles (Figure S1A–D of Supplementary Materials), while nitrate use, IL-6, and hs-cTnI showed a trend to increase (Figure S1E–G of Supplementary Materials).

### 3.2. Plasma HO-1, Coronary Atherosclerosis and Myocardial Ischemia

HO-1 plasma levels were associated with the extent, but not with the severity, of atherosclerosis at coronary CTA (Table 4). In fact, the diagnosis of obstructive CAD and the number of obstructive plaques per patient were similar between HO-1 groups. On the other hand, HO-1 plasma levels were positively related with the extent of global coronary atherosclerotic burden. In fact, the total number of plaques, CAC score, and, in particular, the number of non-obstructive plaques and of calcified plaques were significantly higher in patients in the “high HO-1” group as compared with those in the “low-HO-1” group. 

**Figure 1:**
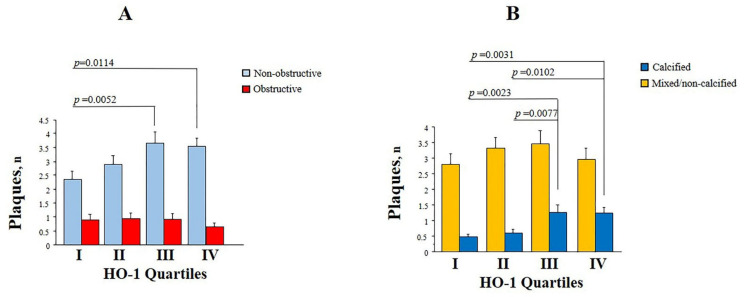
Number of non-obstructive/obstructive plaques (**A**) and calcified/non-calcified-mixed plaques (**B**) according to HO-1 quartiles.

The number of obstructive vs. non-obstructive plaques as well as the number of calcified vs. mixed/non-calcified plaques were compared among HO-1 quartiles (Figure 1). Patients with higher HO-1 plasma levels (III-IV quartiles) showed a significant higher number of non-obstructive plaques when compared with patients with the lowest HO-1 plasma levels (I quartile) (Figure 1A). Moreover, a significantly higher number of calcified plaques was observed in patients with higher HO-1 plasma levels (III-VI quartiles) compared with the others (I-II quartiles) (Figure 1B). The number of obstructive and of mixed/non-calcified plaques was similar among HO-1 quartiles (Figure 1).

Patients in the “high HO-1” group had a higher frequency and a larger extent of myocardial ischemia compared with those in the “low HO-1” group (Table 4).

### 3.3. Plasma HO-1 Levels and Coronary Disease Phenotypes 

**Figure 2:**
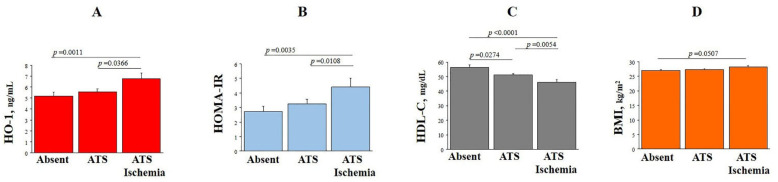

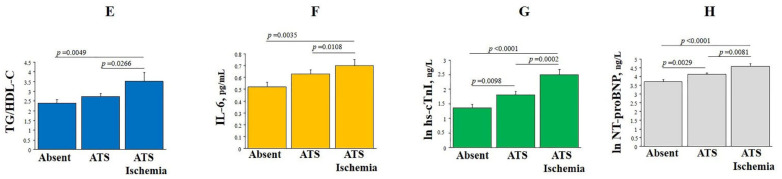
Plasma levels of HO-1 (**A**), HOMA-IR (**B**), HDL-C (**C**), BMI (**D**), TG/HDL (**E**), IL-6 (**F**), and ln transformation of circulating levels of hs-cTnI (**G**), and of NT-proBNP (**H**) in patients divided in groups according to the absence of atherosclerosis and myocardial ischemia, presence of atherosclerosis either alone or combined with myocardial ischemia.

Patients were subdivided into groups according to the absence of atherosclerosis and myocardial ischemia, and the presence of atherosclerosis either alone or combined with myocardial ischemia. Comparison of clinical and bio-humoral features among these groups are reported in Tables S3 and S4 of Supplementary Materials. Patients with coronary atherosclerosis plus ischemia had significantly higher levels of HO-1 as compared with patients without any disease or with only atherosclerosis (Figure 2A). They had also higher levels of HOMA-IR index, BMI, TG/HDL-C ratio and lower of HDL-C (Figure 2B–E) as well as higher levels of IL-6 (Figure 2F), hs-cTnI and NT-proBNP (Figure 2G,H).

## 4. Discussion

**Figure 3:**
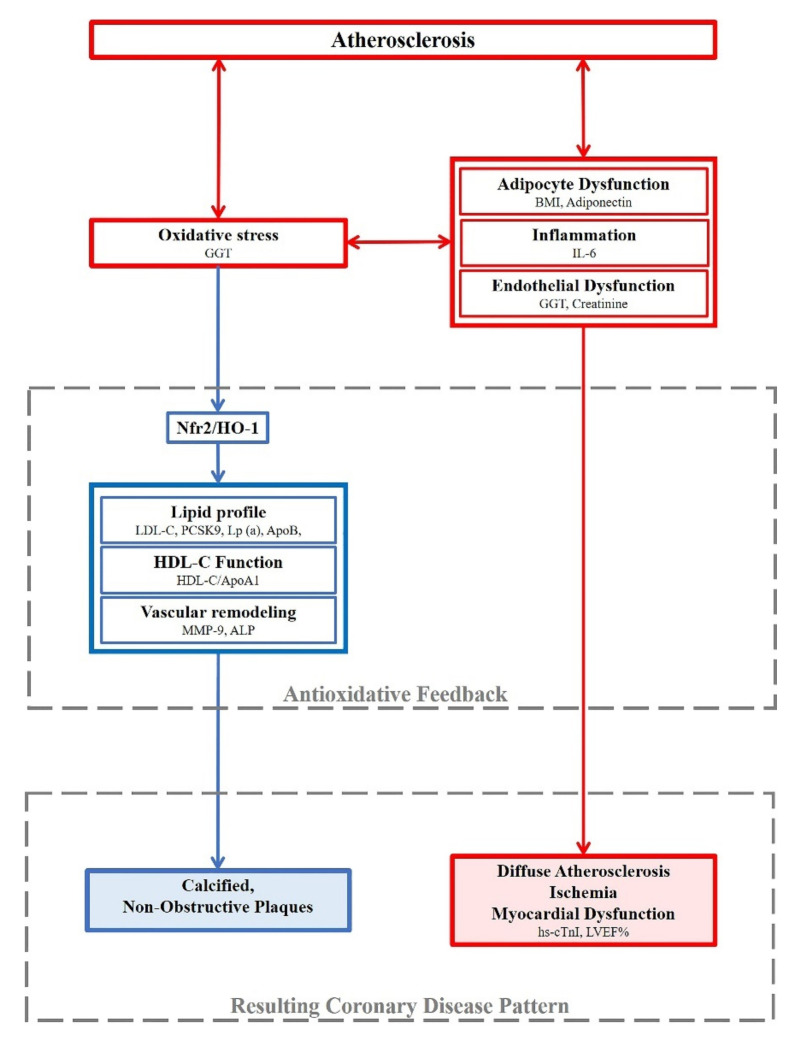
The results of the present study in patients with CCS are framed according to a possible pathophysiologic diagram where “high HO-1” plasma levels reflect a compensatory response to an unbalanced redox state that stimulates Nrf2 activation and HO-1 production. In this context, a higher oxidative stress is putatively linked to adipocyte and endothelial dysfunction, as well as inflammation, all of which are factors that may contribute to the development of atherosclerosis and predispose to ischemia and myocardial dysfunction. On the other hand, higher oxidative stress may stimulate the Nrf2/HO-1 pathway with a potential protective feedback, causing reduction in cholesterol levels, less vascular remodeling, and improvement of cholesterol efflux. The final effect on coronary artery disease pattern would translate into a more diffuse coronary atherosclerosis, mainly with non-obstructive and calcified plaques, together with higher predisposition to myocardial ischemia and dysfunction.

The present study, performed in a well characterized European population of patients with CCS, showed that patients with higher levels of circulating HO-1, a well-known marker of oxidative stress, have a specific clinical phenotype characterized by lower lipid levels, a more diffuse mainly non-obstructive and calcified coronary atherosclerosis, and a higher prevalence of functional ischemia, despite a similar frequency of obstructive disease. These results suggest the potential role of the Nrf2/HO-1 pathway as a protective feedback in clinical coronary disease (Figure 3). 

From a molecular point of view, among the adaptive programs developed by several cell types throughout evolution to counteract oxidative stress, Nrf2/HO-1 activation could be responsible for the favorable CV phenotype observed in patients with higher HO-1 levels by the modulations of processes associated with the metabolic regulation. 

It has been very recently reported that activation of the Nrf2/HO-1 pathway in a hepatic cell line by phytochemical dietary supplementation decreased the expression of two genes involved in cholesterol metabolism, 3-Hydroxy-3-methylglutaryl-CoA reductase (HMGCR), that catalyzes the rate-limiting step in the biosynthesis of cholesterol and is the target of the statin family of drugs, and PCSK9, that can bind to the LDL-C receptor (LDLR), causing LDL-C to be degraded rather than recycled, with the effect of slowing cholesterol removal [21]. Decreased total-C and TG levels were observed also in both serum and liver of ApoE^−/−^ mice and hyperlipidemic golden hamsters after treatment with a Nrf2/HO-1 activator [22]. Thus, these results suggest a role for Nrf2/HO1 in the regulation of cholesterol levels slowing the synthesis and increasing the removal of cholesterol. Of note, the anti-atherogenic effect of statins are partly mediated through HO-1 induction [23,24]. In agreement, in our paper a favorable lipid profile, including significantly lower levels of Total-C, LDL-C, HDL-C, non-HDL-C, Apo B, Lp (a), and PCSK9 was observed in patients with higher HO-1 levels. Of course, there is a strong associative link between high level of serum lipids and the risk of progressive atherosclerosis, since high levels of serum lipid lead to lipid accumulation in the artery wall, which accelerates atherosclerosis [25]. Interestingly, these patients had also significantly lower levels of the HDL-C/Apo A1 ratio, reflecting lower levels of cholesterol-rich HDL particles, which were shown to be associated with preclinical atherosclerosis and mortality [26,27]. In patients with increased HO-1 and decreased cholesterol synthesis, HDL particles are expected to be smaller and relatively depleted of lipids, with increased capacity to accept excess cholesterol from peripheral tissues and thus antagonizing the growing and progression of atherosclerotic plaques [28]. Moreover, activation of the Nrf2/HO1 system attenuates vascular remodeling by decreasing proliferation, migration, and fibrotic processes. These effects are mediated by reduced metalloproteinase activity and decreased protein expression of molecules involved in vascular remodeling [29,30]. Accordingly, in our study, patients with higher HO-1 plasma levels showed lower level of MMP-9 and alkaline phosphatase (ALP). Thus, it is conceivable that all these features, i.e., a favorable lipid profile, a more efficient reverse cholesterol efflux, and inhibition of MMP-9 and ALP production, could explain the presence of lower risk coronary atherosclerosis, characterized by more non-obstructive and calcified plaques observed in patients with high HO-1 plasma levels in this study. Interestingly, this phenotype is similar to what is expected after prolonged statin treatment. In fact, it was recently demonstrated that statins decelerated non-calcified plaque progression, and promoted plaque calcification mainly in patients with non-obstructive CAD [31,32,33]. 

Besides hepatic cells and lipids control, Nfr2/HO-1 signaling plays multiple interacting roles in adipocyte function and obesity-associated metabolic disorders, and controversial data are reported in these pathophysiological processes [34,35]. It has been reported that Nrf2 has a critical role in adipogenesis by regulating the expression of C/EBPb and PPAR [36,37], contributing to the hypertrophy of white adipose tissue and to weight gain [34]. In our study, patients with higher HO-1 plasma levels showed higher BMI values, a higher frequency of metabolic syndrome, higher levels of IL-6, and lower adiponectin. These data are consistent with a condition of adipocyte dysfunction, which is associated with changes of adipocyte-derived paracrine factors, including adipokines and cytokines with potential atherogenic effects. In fact, patients with higher HO-1 plasma levels showed an adipocyte dysfunction-related profile and, in turn, also a more diffuse atherosclerosis with a higher number of plaques, even if mainly non-obstructive and calcific, possibly due to the reduction of cholesterol and statin-like effect discussed previously.

Studies have shown that HO-1 and heme degradation products exert vasodilatory, antioxidant, anti-inflammatory, antiproliferative, and anti-apoptotic effects on vascular cells. Interestingly, these effects are similar, at least in part, to those of eNOS-derived nitric oxide (NO). It has been suggested that in conditions associated with major cardiovascular risk factors (such as obesity, insulin resistance and metabolic syndrome), where endothelial NO production and/or bioavailability is/are decreased and oxidative stress is increased, the Nrf2/HO-1 pathway may be activated to compensate for the loss of NO bioavailability and, at least in part, preserve vascular function [38]. In experimental models of metabolic dysregulation (mimicking obesity and the metabolic syndrome) associated with endothelial dysfunction, pharmacological induction of HO-1 improved cardiovascular function [39,40]. On the other hand, metabolic conditions known to stimulate the Nrf2/HO-1 pathway, can be induced by primary endothelial dysfunction [41].

In our study the subgroup of patients with higher HO-1 plasma levels exhibit a specific metabolic phenotype and, despite a more stable atherosclerotic disease with non-obstructive and calcified plaques, a higher prevalence of inducible ischemia and relatively reduced LV function (Table 1 and Table 3). This observation indicates that higher HO-1 levels might express a protective response to a more pronounced endothelial and vascular dysfunction, as also suggested by higher hs-cTnI, higher creatinine levels and a more frequent use of nitrates.

This possible interplay between HO-1 levels, metabolic/inflammatory and coronary disease phenotype is further evidenced when our population is subdivided according to the absence/presence of coronary atherosclerosis and inducible ischemia (Figure 2). Patients with both atherosclerosis and ischemia, together with higher levels of HO-1, have also significantly altered markers of metabolic syndrome (BMI, HOMA, HDL-C, TG/HDL-C), systemic inflammation (IL-6), ischemic vascular and myocardial dysfunction (hs-cTnI and NTproBNP) [42].

## 5. Conclusions 

The present study demonstrates a previously unknown relationship between plasma HO-1 levels and a bio-humoral and imaging coronary phenotype mainly characterized by reduced cholesterol and a more diffuse coronary atherosclerosis, but with mainly non-obstructive and calcified plaques. The overall outcome from in vitro and preclinical studies claimed a role for HO-1 as potential therapeutic target in ASCVD. In fact, a number of natural antioxidant compounds contained in foods and plants, such as curcumin and caffeic acid phenethyl ester (polyphenols), and sulforaphane (isothiocyanates), have been demonstrated to be effective inducers of HO-1 and exert defensive actions against oxidative stress-related diseases [43,44,45]. However, a full understanding of adipocyte function and obesity-associated metabolic disorders and of the multiple interacting roles of Nrf2/HO-1 signaling in these patho-physiological processes will require further investigations. Research aiming to target in more depth the link between Nrf2/HO-1 pathway, endothelial function, and adipocyte function is much needed. From a clinical perspective, the new information gathered on the interaction of Nrf2/HO-1 pathway with lipid metabolic status, adipose and endothelial function, inflammation and the atherosclerotic phenotype might be useful to develope new targeted individual treatments in the context of a personalized medicine approach.

## Figures and Tables

**Table 1:** Clinical characteristics of the whole population relative to HO-1 levels.

	Clinical Population *n* = 526	Low HO-1 *n* = 263	High HO-1 *n* = 263	*p* Value
**Demographics**				
Age, years	60 ± 9	61 ± 8	60 ± 9	ns
Males	318 (60)	145 (55)	173 (66)	0.0125
**Clinical characteristics**				
Typical angina	139 (26)	76 (29)	63 (24)	ns
LVEF%	60 ± 8	61 ± 8	59 ± 8	0.0110
CAD probability	48 ± 19	48 ± 20	49 ± 18	ns
**Cardiovascular risk factors**				
Family history of CAD	186 (35)	94 (36)	92 (37)	ns
Diabetes	177 (34)	83 (32)	94 (36)	ns
Hypercholesterolemia	316 (60)	157 (60)	159 (60)	ns
Hypertension	349 (66)	166 (63)	163 (62)	ns
Smoking	129 (24)	64 (24)	65 (25)	ns
BMI, kg/m^2^	27.7 ± 4.3	27.2 ± 4	28.2 ± 4.6	0.0076
Metabolic syndrome	181 (34)	80 (30)	101 (38)	0.0539
**Pharmacological therapies**				
Beta-blockers	212 (40)	100 (38)	112 (43)	ns
Calcium channel blockers	72 (14)	30 (11)	42 (16)	ns
ACE Inhibitors	157 (30)	86 (33)	71 (27)	ns
ARBs	89 (17)	41 (16)	48 (18)	ns
Diuretics	88 (17)	44 (17)	44 (17)	ns
Anti-diabetic	109 (21)	45 (17)	51 (19)	ns
Statins	274 (52)	128 (49)	146 (56)	ns
Aspirin	309 (59)	155 (59)	154 (59)	ns
Nitrates	58 (11)	23 (9)	35 (13)	ns
Anti-coagulants	11 (2)	4 (1)	7 (3)	ns

Continuous variables are presented as mean ± standard deviation, categorical variables as absolute N and (%).

**Table 2:** Bio-humoral characteristics of the whole population relative to HO-1 levels.

	Clinical Population *n* = 526	Low HO-1 *n* = 263	High HO-1 *n* = 263	*p* Value
**Oxidative stress**				
HO-1, ng/mL	5.65 ± 4.19	2.33 ± 1.43	8.97 ± 3.13	<0.0001
GGT, IU/L	40 ± 30	38 ± 27	42 ± 32	0.0074
**Metabolic (glucose)**				
FPG, mg/dL	112 ± 36	112 ± 34	113 ± 38	ns
Insulin, μUI/mL	11.6 ± 11.0	12.7 ± 10.7	11.5 ± 11	ns
HOMA-IR index	3.5 ± 4.2	3.4 ± 4.1	3.5 ± 4.1	ns
**Metabolic (lipid)**				
Total-C, mg/dL	183 ± 49	188 ± 50	178 ± 49	0.0166
LDL-C, mg/dL	106 ± 40	110 ± 40	102 ± 40	0.0130
HDL-C, mg/dL	52 ± 17	54 ± 16	51 ± 18	0.0054
Remnant-C, mg/dL	24 ± 15	23 ± 14	25 ± 15	ns
Non-HDL-C, mg/dL	130 ± 43	134 ± 43	126 ± 42	0.0528
Apo A1, mg/dL	143 ± 32	145 ± 33	142 ± 32	ns
HDL-C/Apo A1	0.37 ± 0.12	0.38 ± 0.13	0.36 ± 0.1	0.0258
Apo B, mg/dL	87 ± 28	90 ± 28	84 ± 28	0.0079
Apo A1/Apo B	1.80 ± 0.85	1.71 ± 0.52	1.89 ± 1.07	0.0167
Lp (a)	20 ± 22	23.6 ± 24.1	18.6 ± 21.8	0.0076
Triglycerides, mg/dL	124 ± 81	120 ± 75	128 ± 86	ns
TG/HDL-C	2.74 ± 2.45	2.55 ± 2.33	2.94 ± 2.55	ns
PCSK9, ng/mL	213 ± 105	227 ± 110	199 ± 98	0.0024
**Adipose tissue**				
Adiponectin, μg/mL	9.8 ± 6.9	10.2 ± 6.4	9.2 ± 6.9	0.0030
Leptin, ng/mL	9.9 ± 10.7	8.8 ± 8.3	11.2 ± 12.7	ns
**Hepatic**				
AST, IU/L	24 ± 10	24 ± 9	25 ± 11	ns
ALT, IU/L	21 ± 13	20 ± 13	22 ± 14	ns
**Remodeling**				
MMP-2, ng/mL	159 ± 61	157 ± 63	161 ± 58	ns
MMP-9, ng/mL	145 ± 206	162 ± 221	127 ± 187	0.0511
ALP, IU/L	51 ± 18	53 ± 18	50 ± 18	0.0433
**Inflammatory**				
hs-CRP, mg/dL	0.40 ± 1.09	0.34 ± 0.56	0.47 ± 1.45	ns
IL-6, ng/L	1.35 ± 2.35	1.12 ± 1.33	1.47 ± 2.83	0.0575
**Cardiac**				
hs-cTnT, ng/L	910 ± 21	10 ± 19	10 ± 22	ns
hs-cTnI, ng/L	54 ± 240	41 ± 215	66 ± 262	0.0006
NT-proBNP, ng/L	139 ± 291	119 ± 173	158 ± 374	ns
**Renal**				
Creatinine, mg/dL	0.96 ± 0.23	0.85 ± 0.23	0.91 ± 0.22	0.0014

Continuous variables are presented as mean ± standard deviation.

**Table 3:** Univariate and multivariate analysis.

	Univariate			Multivariate		
	Coefficient	SE	*p* Value	Coefficient	SE	*p* Value
Age	−0.010	0.004	0.0218	−0.011	0.004	0.0128
Males	0.243	0.079	0.0022			
LVEF%	−0.010	0.005	0.0286			
BMI	0.025	0.009	0.0068			
Beta-blockers	0.141	0.079	0.0748			
Statins	0.198	0.078	0.0108			
Nitrates	0.249	0.124	0.0452	0.267	0.124	0.0317
GGT	0.173	0.072	0.0159			
Total-C	−0.466	0.143	0.0012			
LDL-C	−0.289	0.095	0.0026			
HDL-C	−0.394	0.122	0.0013			
Non-HDL-C	−0.002	0.001	0.0065			
HDL-C/Apo A1	−1.214	0.332	0.0003	−1.281	0.343	0.0002
Apo B	−0.369	0.117	0.0145	−0.390	0.126	0.0017
Apo A1/Apo B	0.111	0.046	0.0163			
Lp (a)	−0.116	0.038	0.0024	−0.101	0.038	0.0087
PCSK9	−0.306	0.080	0.0002			
Adiponectin	−0.186	0.061	0.0024			
MMP-9	−0.063	0.037	0.0876			
ALP	−0.284	0.108	0.0088			
IL-6	0.244	0.081	0.0027	0.180	0.081	0.0274
hs-cTnI	0.093	0.027	0.0005	0.064	0.027	0.0189

**Table 4:** Imaging results relative to HO-1 groups.

	Imaging Population *n* = 347	Low HO-1 *n* = 174	High HO-1 *n* = 173	*p* Value
**Coronary Anatomy**				
Normals	95 (27)	52 (30)	42 (24)	ns
Patients with non-obstructive	131 (38)	59 (34)	72 (42)	
Patients with obstructive	121 (35)	62 (36)	59 (34)	
**Coronary Plaques**				
Total No. of plaques	4 ± 3.8	3.6 ± 3.5	4.5 ± 4	0.0265
No. of non-obstructive plaques	3.1 ± 3	2.6 ± 2.6	3.6 ± 3.3	0.0018
No. of obstructive plaques	0.9 ± 14.7	0.9 ± 1.8	0.8 ± 1.5	ns
No. of calcified plaques	0.9 ± 1.7	0.5 ± 1	1.2 ± 2.1	0.0002
No. of non-calcified plaques	0.5 ± 0.9	0.4 ± 0.9	0.5 ± 0.9	ns
No. of mixed plaques	2.7 ± 3.2	2.6 ± 3.1	2.83 ± 3.4	ns
**Risk Scores**				
CTA risk score	11.9 ± 11	11 ± 10.6	12.8 ± 11.3	ns
CAC score (*n* = 286)	292 ± 604	222 ± 414	361 ± 739	0.0497
**Myocardial Ischemia**				
Patients with myocardial ischemia	83 (24)	34 (20)	49 (28)	0.0477
SDS at MPI (*n* = 274)	3.41 ± 7.71	2.19 ± 5.12	4.14 ± 8.78	0.0272

## Data Availability

The data presented in this study are available in article and Supplementary Materials.

## Supplementary Materials

The following are available online at https://www.mdpi.com/article/10.3390/antiox10122002/s1, Table S1: Effect of clinical variables on HO-1 plasma levels at univariate analysis, Table S2: Effect of bio-humoral variables on HO-1 plasma levels at univariate analysis, Table S3: Clinical features of imaging population according to absence/presence of atherosclerosis, with or without ischemia; Table S4: Bio-humoral profile of imaging population according to absence/presence of atherosclerosis, with or without ischemia, Figure S1: Distribution of independent predictors of HO-1 plasma levels (at multivariate analysis) according to HO-1 quartiles.
