# Supplementary material for: Association of Circulating Heme Oxygenase-1, Lipid Profile and Coronary Disease Phenotype in Patients with Chronic Coronary Syndrome
Source: Antioxidants (Basel). 2021 Dec 15;10(12):2002. doi: 10.3390/antiox10122002 (PMC8698632; doi:10.3390/antiox10122002)
Supplement: Supplementary file 1 [file antioxidants-10-02002-s001.zip › antioxidants-1501517-supplementary.pdf]

Supplemental material

Table S1. Effect of clinical variables on HO-1 plasma levels at univariate analysis

	coefficient	SE	P value
**Demographics**			
Age, years	-0.010	0.004	0.0218
Males	0.243	0.079	0.0022
**Clinical characteristics**			
Typical angina	-0.069	0.088	ns
LVEF%	-0.010	0.005	0.0286
CAD probability	0.002	0.002	ns
**Cardiovascular risk factors**			
Family history of CAD	0.013	0.082	ns
Diabetes	0.121	0.082	ns
Hypercholesterolemia	0.032	0.80	ns
Hypertension	0.030	0.083	ns
Smoking	0.042	0.091	ns
BMI, kg/m^2	0.025	0.009	0.0068
Metabolic Syndrome	0.133	0.082	0.1044
**Pharmacological therapies**			
Beta-blockers	0.141	0.079	0.0748
Calcium channel blockers	0.097	0.113	ns
ACE Inhibitors	-0.052	0.085	ns
ARBs	-0.33	0.104	ns
Diuretics	-0.061	0.104	ns
Anti-diabetic	0.021	0.096	ns

Statins	0.198	0.078	0.0108
Aspirin	0.017	0.079	ns
Nitrates	0.249	0.124	0.0452
Anti-coagulants	0.391	0.272	ns

Table S2. Effect of bio-humoral variables on HO-1 plasma levels at univariate analysis

Biomarkers	coefficient	SE	P value
**Oxidative stress**			
GGT, IU/L	0.173	0.072	0.0159
**Metabolic (glucose)**			
FPG, mg/dL	0.073	0.150	ns
Insulin, μUI/mL	0.019	0.059	ns
HOMA-IR index	0.044	0.068	ns
**Metabolic (lipid)**			
Total-C, mg/dL	-0.466	0.143	0.0012
LDL-C, mg/dL	-0.289	0.095	0.0026
HDL-C, mg/dL	-0.394	0.122	0.0013
Remnant-C, mg/dL	0.002	0.003	ns
Non-HDL-C, mg/dL	-0.002	0.001	0.0065
Apo A1, mg/dL	-0.054	0.154	ns
HDL-C/Apo A1	-1.214	0.332	0.0003
Apo B, mg/dL	-0.278	0.113	0.0145
Apo A1/Apo B	0.111	0.046	0.0163
Lp (a)	-0.116	0.038	0.0024
TG, mg/dL	0.037	0.072	ns
TG/HDL-C	0.020	0.016	ns
PCSK9, ng/mL	-0.306	0.080	0.0002
**Adipose Tissue**			
Adiponectin, μg/mL	-0.186	0.061	0.0024
Leptin, ng/mL	0.058	0.045	ns

**Hepatic**			
AST, IU/L	0.053	0.108	ns
ALT, IU/L	-0.005	0.081	ns
**Remodeling**			
MMP-2, ng/mL	-0.035	0.092	ns
MMP-9, ng/mL	-0.063	0.037	0.0876
ALP, IU/L	-0.284	0.108	0.0088
**Inflammatory**			
hs-CRP, mg/dL	0.195	0.122	ns
IL-6, ng/L	0.244	0.081	0.0027
**Cardiac**			
hs-cTnT, ng/L	0.0001	0.059	ns
hs-cTnI, ng/L	0.093	0.027	0.0005
NT-proBNP, ng/L	0.007	0.033	ns
**Renal**			
Creatinine, mg/dL	1.213	0.333	0.0003

Figure S1. Distribution of independent predictors of HO-1 plasma levels (at multivariate analysis) according to HO-1 quartiles.

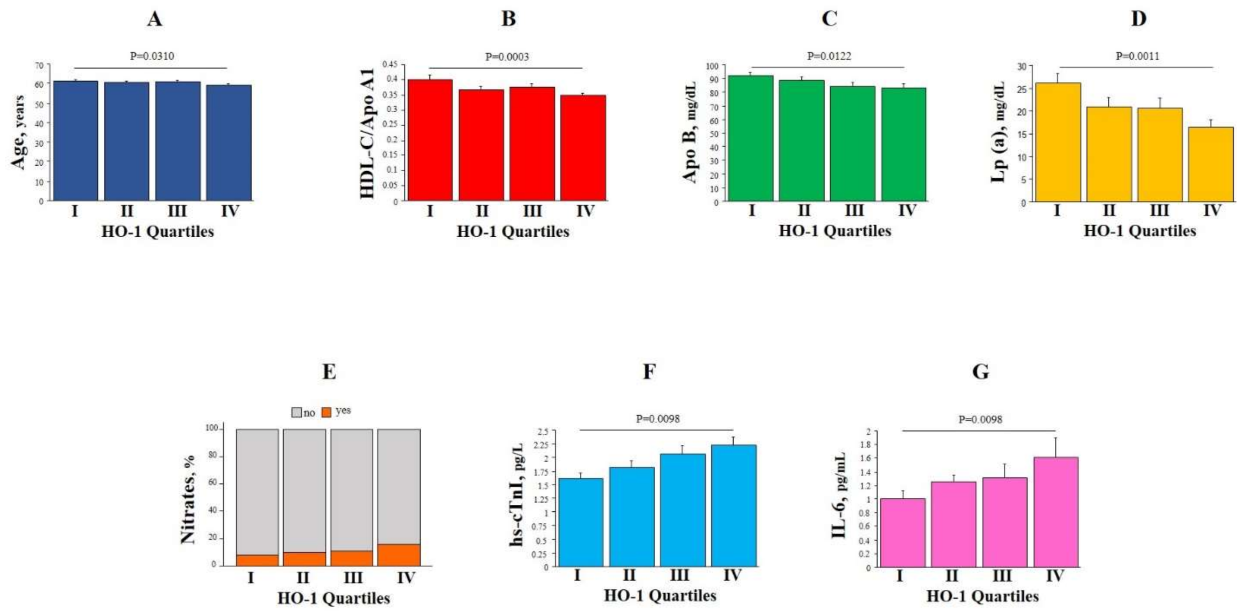

Table S3. Clinical features of imaging population according to absence/presence of atherosclerosis, with or without ischemia.

	Imaging population n=347	Absent n=97	Atherosclerosis n=167	Atherosclerosis/ Ischemia n=83	P value
**Demographics**					
Age, years	60±9	55±8	62±8	63±8	<0.0001
Males	211 (61)	43 (44)	103 (62)	65 (78)	<0.0001
**Clinical characteristics**					
Typical angina	78 (22)	23 (24)	29 (17)	26 (31)	0.0425
LVEF%	60±8	61±7	61±8	56±8	<0.0001
CAD probability	47±19	39±18	47±17	56±21	<0.0001
**CVD risk factors**					
Family history of CAD	117 (34)	40 (41)	64 (38)	13 (16)	0.0003
Diabetes	103 (30)	24 (25)	46 (27)	33 (40)	ns
Hypercholesterolemia	202 (58)	50 (52)	103 (62)	49 (59)	ns
Hypertension	210 (61)	43 (44)	118 (71)	49 (59)	0.0001
Smoking	82 (2421)	20 (30)	42 (25)	20 (27)	ns
BMI, kg/m^2	27.4±3.9	26.9±3.9	27.4±3.8	28.2±4.2	ns
Metabolic Syndrome	125 (36)	34 (35)	59 (35)	32 (39)	ns
**Pharmacological therapies**					
Beta-blockers	142 (41)	32 (33)	69 (41)	41 (49)	ns
Calcium channel blockers	43 (12)	9 (9)	18 (11)	16 (19)	ns
ACE Inhibitors	96 (28)	19 (20)	51 (31)	26 (31)	ns
ARBs	55 (16)	9 (9)	34 (20)	12 (15)	ns

Diuretics	59 (17)	13 (13)	33 (20)	13 (16)	ns
Anti-diabetic	58 (17)	9 (9)	29 (17)	20 (24)	0.0280
Statins	183 (53)	37 (38)	92 (55)	54 (54)	0.0011
Aspirin	202 (58)	42 (43)	99 (59)	61 (71)	0.0002
Nitrates	37 (11)	9 (6)	14 (8)	17 (20)	0.0034
Anti-coagulants	7 (2)	2 (2)	2 (1)	3 (4)	ns

Table S4. Bio-humoral profile of imaging population according to absebce/presence of atherosclerosis, with or without ischemia.

	Imaging population n=347	Absent n=97	Atherosclerosis n=167	Atherosclerosis/ Ischemia n=83	P value
**Oxidative stress**					
HO-1, ng/mL	5.70±4.09	5.16±3.67	5.57±4.01	6.80±4.56	0.0314
GGT, IU/L	40±31	41±32	37±19	45±45	ns
**Metabolic (glucose)**					
FPG, mg/dL	111±34	108±27	110±37	116±36	ns
Insulin, μUI/mL	11.5±11.3	9.7±8.6	10.9±10.5	14.5±14.8	0.0149
HOMA-IR index	3.4±4.2	2.8±3.3	3.2±3.9	4.4±5.3	0.0085
**Metabolic (lipid)**					
Total-C, mg/dL	180±47	192±47	178±48	168±42	0.0053
LDL-C, mg/dL	105±39	113±38	104±41	97±34	0.0376
HDL-C, mg/dL	51±16	56±19	51±14	46±15	<0.0001
Remnant-C, mg/dL	24±15	23±12	25±16	24±15	ns
Non-HDL-C, mg/dL	129±42	135±40	128±43	122±40	ns
Apo A1, mg/dL	143±33	152±32	141±33	137±34	0.0057
HDL-C/Apo A1	0.37±0.12	0.37±0.11	0.38±0.14	0.35±0.11	ns
Apo B, mg/dL	87±27	88±23	87±28	85±29	ns
Apo A1/Apo B	1.77±0.59	1.82±0.58	1.75±0.54	1.76±0.68	ns
Lp (a)	21±23	19±21	22±25	22±23	ns
TG, mg/dL	124±78	115±61	125±80	131±91	ns
TG/HDL-C	2.81±2.63	2.39±1.93	2.72±2.17	3.50±3.81	0.0153

PCSK9, ng/mL	212±107	225±118	216±112	190±77	ns
**Adipose Tissue**					
Adiponectin, µg/mL	9.2±6.2	9.2±5.3	9.2±6.4	9.3±6.7	ns
Leptin, ng/mL	10.1±10.8	12.3±12.5	9.6±10.8	8.3±8.1	0.0248
**Hepatic**					
AST, IU/L	24±10	24±11	23±9	26±11	0.0393
ALT, IU/L	21±13	22±17	20±11	22±11	ns
**Remodeling**					
MMP-2, ng/mL	160±60	154±60	160±61	167±58	ns
MMP-9, ng/mL	136±204	125±157	141±206	121±211	ns
ALP, IU/L	50±17	50±18	50±17	50±17	ns
**Inflammatory**					
hs-CRP, mg/dL	0.34±0.59	0.33±0.72	0.30±0.46	0.41±0.69	ns
IL-6, ng/L	1.04±1.14	0.82±0.90	1.06±1.22	1.27±1.39	0.0111
**Cardiac**					
hs-cTnT, ng/L	8±6	6±4	8±5	9±8	<0.0001
hs-cTnI, ng/L	51±249	25±148	51±287	80±260	<0.0001
NT-proBNP, ng/L	115±166	71±67	109±160	178±230	<0.0001
**Renal**					
Creatinine, mg/dL	0.86±0.21	0.86±0.23	0.85±0.19	0.87±0.22	ns
